# Analyzing the expression of candidate microRNAs in primary tumors of oral squamous cell carcinoma

**DOI:** 10.1186/1755-8166-7-S1-P7

**Published:** 2014-01-21

**Authors:** Mayakannan Manikandan, Deva Magendhra Rao A K, Arasambattu Kannan Munirajan

**Affiliations:** 1 Department of Genetics, Dr. ALM PG Institute of Basic Medical Sciences, University of Madras, Taramani campus, Chennai – 600113, Tamil Nadu, India

## Background

Accumulating evidences suggest that aberrant expression of microRNAs (miRNAs) contribute to the initiation, development, invasion and metastasis of human cancers including Oral Squamous Cell Carcinoma (OSCC).

## Materials and methods

In this study, we investigated the differential expression of six candidate miRNAs namely hsa-miR-21, hsa-miR-125b-2, hsa-miR-138, hsa-miR-155, hsa-miR-184 and hsa-miR-205 in thirty two OSCC primary tumors in comparison to five normal control tissue samples by employing TaqMan® MicroRNA Assays.

## Results

Amongst the studied candidates, miR-125b-2 and miR-205 were significantly down regulated while miR-21 and miR-155 were significantly upregulated in the primary tumors compared to controls. The other two miRNAs, miR-184 and miR-138, in general were down regulated in the cancer samples although not statistically significant. These observations suggest that microRNA dysfunction could be one among the major factors for initiation and progression of oral cancer with miR-125b-2 and miR-205 functioning as tumor suppressor miRs, and miR-155 and mir-21 acting as oncomiRs. Functional annotation of the experimentally validated targets of these miRNAs highlighted that miR-21, miR-125b-2 and miR-155 were predominantly targeting genes of the apoptotic pathway, while miR-205 was targeting genes involved in transcriptional and metabolic processes.

## Conclusions

It is concluded that microRNA dysfunction could be one among the major factors for initiation and progression of oral cancer. However, further experimental studies in more OSCC primary tumors are required to facilitate the use of these miRNAs as molecular biomarkers or as diagnostic/prognostic indicators.

